# Oxidative stress parameters in women and men with suicidal thoughts and following a suicide attempt

**DOI:** 10.3389/fpsyt.2024.1382303

**Published:** 2024-04-24

**Authors:** Magdalena Lech, Lucyna Ostrowska, Napoleon Waszkiewicz, Agnieszka Kułak-Bejda, Mateusz Maciejczyk, Katarzyna Witczak-Sawczuk, Anna Zalewska, Karolina Dańkowska, Małgorzata Żendzian-Piotrowska

**Affiliations:** ^1^ Department of Dietetics and Clinical Nutrition Medical University of Bialystok, Bialystok, Poland; ^2^ Department of Psychiatry, Medical University of Bialystok, Bialystok, Poland; ^3^ Department of Hygiene, Epidemiology and Ergonomics Medical University of Bialystok, Bialystok, Poland; ^4^ Department of Conservative Dentistry, Medical University of Bialystok, Bialystok, Poland; ^5^ Students’ Scientific Club “Biochemistry of Civilization Diseases” at the Department of Hygiene, Epidemiology and Ergonomics, Medical University of Bialystok, Bialystok, Poland

**Keywords:** oxidative stress, suicidal, human disease, depression, schizophrenia, psychiatry

## Abstract

**Background:**

This study aimed to evaluate oxidative stress parameters in individuals with depression and schizophrenia, considering gender differences, and manifesting suicidal behavior, encompassing thoughts without a tendency to be realized, thoughts with a tendency to be realized, and suicide attempts.

**Methods:**

From among the patients from Department of Psychiatry 120 individuals were selected who met the inclusion criteria and did not meet the exclusion criteria for the study. In the initial phase of the project, patients eligible for the study underwent the M.I.N.I 7.0.2 questionnaire (Mini International Neuropsychiatric Interview). Subsequently, in the second phase of the research, venous blood samples were collected from the patients for the purpose of conducting biochemical assessments, focusing on oxidative stress parameters.

**Results:**

The obtained results suggest that redox biomarkers, namely TOS (total oxidation state) and OSI (TOS/TAC ratio), in the blood plasma of women increase in tandem with the severity of suicidal behavior. No notable alterations in SOD (Cu-Zn-superoxide dismutase), GPx (glutathione peroxidase), and GSH (reduced glutathione) concentrations and activity were noted between groups exhibiting suicidal behavior. The observed variations in the concentrations and activity of antioxidant parameters were significant solely in comparison to the control group.

**Conclusions:**

Redox biomarkers TOS and OSI could prove valuable in diagnosing women at a genuine risk of committing suicide. On the other hand, antioxidant parameters – SOD, GPx, and GSH may be instrumental in identifying patients with suicidal behaviors, without specifying their intensity.

## 1 Introduction

Suicide involves intentional bodily harm with the intention of causing death. Suicidal behavior encompasses thoughts about committing suicide, including those with a plan for implementation and those that occur without a specific plan. The majority of individuals attempting suicide are psychiatric patients (1). Approximately 21% of individuals diagnosed with schizophrenia attempt suicide, while 39% of those with unipolar and bipolar depression make such attempts (2, 3). It is acknowledged that not all suicidal thoughts lead to actual attempts (4). However, the presence of suicidal thoughts is recognized as a significant risk factor for attempting suicide. The number of suicidal thoughts significantly surpasses the number of attempts made by individuals (3). It is critical to highlight that suicidal behaviors, including thoughts without a tendency to be realized, thoughts with a tendency to be realized, and suicide attempts, constitute a complex process. Individuals attempting suicide usually have previously experienced both types of thoughts, which means they were unable to seek adequate assistance (5, 6). There is a need for precise methods to differentiate individuals at risk of attempting suicide among those reporting such thoughts (3). Scientific research suggests a relationship between the presence and intensity of suicidal behavior and changes in the concentration of certain biochemical parameters, including oxidative stress and inflammatory factors, in the body (5). Although the significance of these disorders in chronic mental diseases is emphasized, the role of oxidative stress in psychiatry remains unclear (5).

Oxidative stress (OS) occurs when there is an excess production of reactive oxygen species (ROS) or reactive nitrogen species (RNS), leading to a diminished antioxidant capacity in the body (3, 4). OS is implicated in causing cell and DNA damage (4). Research indicates a connection between inflammatory neurotoxicity, nitrooxidative stress, and suicidal behavior. The brain, being an organ with high oxygen consumption vital for the functioning of the central nervous system (CNS), is particularly vulnerable to oxidative stress (7). Depression and schizophrenia are prevalent chronic mental disorders often associated with frequent hospitalizations. Numerous studies suggest that individuals post-hospitalization are 28 to 49% more likely to attempt suicide (6). Depression is linked to oxidative stress parameters, evident through the presence of markers for lipid peroxidation and inadequate antioxidant protection (8). Growing evidence highlights the association between inflammation and schizophrenia, consequently indicating the presence of oxidative stress parameters. Individuals undergoing treatment for schizophrenia exhibit reduced levels of glutathione (GSH), superoxide dismutase (SOD), and catalase (CAT) (9). Neurochemical changes occurring during psychosis trigger the OS process, leading to the overactivation of NMDA (N-methyl-D-aspartate receptor) glutamate cells and altering the function of dopamine receptors (9).

In light of recent scientific findings, the examination of oxidative stress markers in psychiatric patients exhibiting suicidal tendencies appears to be warranted. Consequently, this study aimed to evaluate oxidative stress parameters in individuals with depression and schizophrenia, considering gender differences, and manifesting suicidal behavior, encompassing thoughts without a tendency to be realized, thoughts with a tendency to be realized, and suicide attempts. Realistically assessing the risk of suicide attempts is crucial for providing effective assistance to patients suffering from mental illness, ultimately aiming to reduce the mortality rate due to suicide in this population. Targeted efforts directed at individuals within the high-risk group represent one of the key avenues to mitigate the incidence of suicide (6).

## 2 Materials and methods

### 2.1 Study group

**Figure 1:**
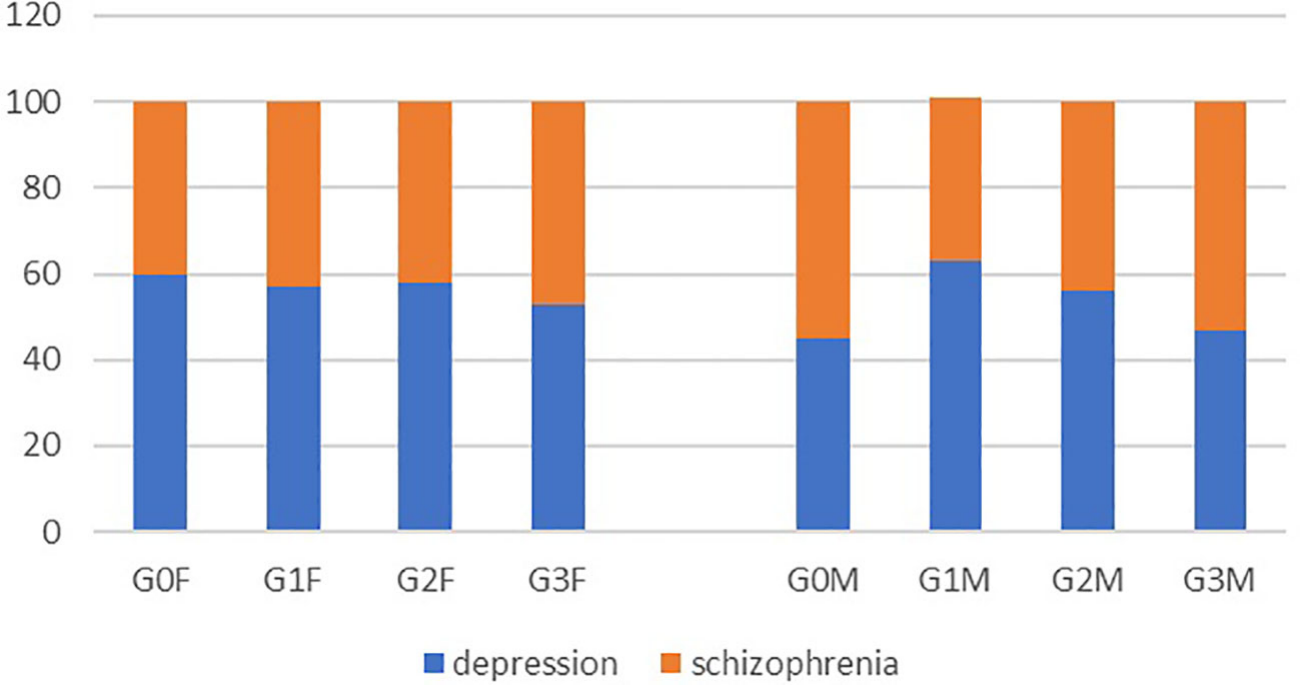
Characteristics of the examined patients in terms of diagnosis (depression and schizophrenia) in respective groups.

The study involved patients admitted to the Department of Psychiatry at the Medical University of Bialystok due to mental deterioration and diagnosed with unipolar depression or schizophrenia. From among the patients hospitalized in the ward, 120 individuals were selected who met the inclusion criteria and did not meet the exclusion criteria for the study. The inclusion criteria encompassed a diagnosis of depression or schizophrenia (see **Figure 1**) and patients aged between 20 and 50 years. Exclusion criteria included the use of psychoactive substances, the coexistence of central neurological system diseases (stroke, dementia, intellectual disability) and other severe somatic conditions. Patients included in the study were not receiving lithium treatments but were taking antipsychotic and antidepressant medications (olanzapine, aripiprazole, chlorpromazine, chlorprothixene, promazine, zuclopenthixol, quetiapine, risperidone, mirtazapine, sertraline, trazodone, venlafaxine, citalopram, sulpiride, mianserin, moclobemide, duloxetine, haloperidol). In the initial phase of the project, patients eligible for the study underwent the M.I.N.I 7.0.2 questionnaire (part B – suicidal thoughts, self-harm, suicidal behavior). Based on the obtained results, patients were categorized into specific groups (**Table 1**). The assessment was conducted within the first 48 hours of the patient’s admission to the ward. In a cohort of 120 patients admitted to the hospital for depression or schizophrenia, the participants were categorized into eight groups: females without suicidal tendencies (G0F), females with suicidal thoughts without the tendency to act on them (G1F), females with suicidal thoughts with the tendency to act on them (G2F), females post suicide attempt (G3F), males without suicidal tendencies (G0M), males with suicidal thoughts without the tendency to act on them (G1M), males with suicidal thoughts with the tendency to act on them (G2M), and males post suicide attempt (G3M). Oxidative and antioxidant parameters related to oxidative stress were assessed in this study. 47.5% (n=57) of all the patients were women, while 52.5% (n=63) were men.

**Table 1:** Division of the subjects into respective groups.

GROUP DESIGNATION	M.I.N.I 7.0.2 SCORE	GROUP IDENTIFICATION	SEX	n	Total n
No suicidal tendencies	0 points	G0F	WOMEN	10	30
		G0M	MEN	20	
Suicidal thoughts lacking the tendency to act on them	1-8 points	G1F	WOMEN	14	30
		G1M	MEN	16	
Suicidal thoughts with the tendency to act on them	9-16 points	G2F	WOMEN	19	28
		G2M	MEN	9	
Post suicide attempt	≥17 points	G3F	WOMEN	15	32
		G3M	MEN	17	

Subsequently, the presentation of the characteristics of the examined patients was provided, focusing on the diagnosis of the disease (depression, schizophrenia) within each group (**Figure 1**).

**Figure 2:**
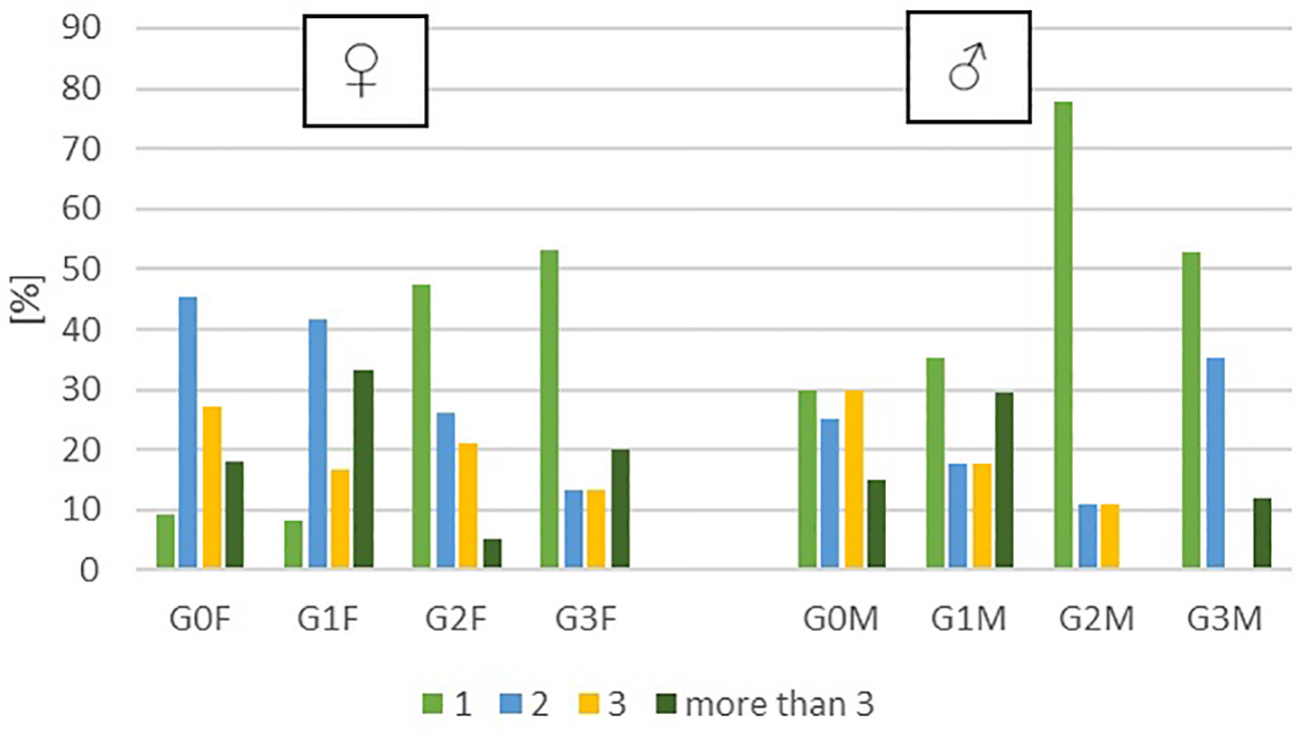
The number of hospitalizations in women and men in respective groups.

The analysis of the frequency of hospitalizations among the patients participating in the study is illustrated in **Figure 2**.

The majority of individuals assigned to the G3F and G3M categories made their suicide attempt for the first time (73% of female patients and 82% of male patients). There were no statistically significant differences between the groups in terms of the number of hospitalizations. Subsequently, in the second phase of the research, venous blood samples were collected from the patients for the purpose of conducting biochemical assessments, focusing on oxidative stress parameters.

### 2.2 Methodology of assessment

Blood samples for the study were collected within the first 48 hours of hospitalization, following a MINI 7.0.2 interview. Venous blood required for assessing the concentration (TOS, OSI, GSH, TAC, FRAP) and activity (GPx, SOD) of oxidative stress parameters was collected into K-3 EDTA s-monovette tubes in the collection room after an overnight fast. The tubes were labeled and coded for the study, then centrifuged at 3000 xg for 20 minutes at 4°C (MPV-351R). After plasma separation, the samples were stored at -80°C in Eppendorf tubes until assays were performed. The analyses were conducted utilizing a Sigma-Aldrich device with a 96-well microplate reader, and the absorbance/fluorescence of the plates was measured. The results were standardized to 1 mg of proteins, and a dual-sample approach was employed to validate the findings. The assessments were carried out at the Department of Hygiene, Epidemiology, and Ergonomics of the Medical University of Białystok.

The following oxidative stress metabolites were analyzed:

- TOS (total oxidation state – nmol H_2_O_2_ Equiv/mg protein) was assessed bichromatically by inducing the oxidation of Fe^2+^ to Fe^3+^ in the presence of oxidants within the sample (560/800 nm) (10);
- OSI (TOS/TAC ratio) was computed using the OSI = TOS/TAC formula (11);
- GPx (glutathione peroxidase – mU/mg protein) was determined by measuring NADPH coupled with the reduction of GSSH (12);
- SOD (Cu-Zn-superoxide dismutase - mU/mg protein) was assessed by gauging the inhibition rate of adrenaline oxidation (480 nm) (13);
- GSH (reduced glutathione - µg/mg protein) was derived from the standard curve for GSH solutions and expressed as g/mg protein, with sample absorbance at 412 nm (14);
- TAC (umol/mg protein) was assessed spectrophotometrically, wherein the TAC concentration was determined using the radical cation ABTS (2,2-azinobis-3-ethylbenzothiazoline-6-sulfonic acid) and Trolox (6-hydroxy-2,5,7,8 acid -tetramethylchroman-2-carboxylic acid) – at a wavelength of 660 nm (15);
- FRAP (umol/mg protein) was assessed colorimetrically. The formation of the colored iron complex tripyridyltriazine (Fe^3+-^TPTZ) resulted from the reduction of Fe^2+^ to Fe^3+^. The reaction occurred in an acidic medium with an absorbance of 592 nm (16);

The examination of the concentration and activity of specific parameters revealed statistically significant variances for the following markers: TOS, OSI, SOD, GSH, TAC, FRAP, and GPx within the entire cohort of subjects.

### 2.3 Statistical analysis

The study results were statistically analyzed using the STATISTICA 13.0 test and the R-project test. Statistical significance was considered at p<0.05. The normality of distribution was assessed using the Shapiro-Wilk test. To examine the hypothesis of the insignificance of differences between the medians of the studied variable in individual groups, the Kruskal-Wallis ANOVA test was employed. For a more detailed analysis, Dunn’s post-hoc test was utilized to compare the differences between groups.

## 3 Results

The evaluation of the levels of oxidative stress factors in the blood plasma of the subjects is outlined in **Table 2**.

**Table 2:** Results of oxidative stress parameters in the blood plasma of the examined patients.

oxidative stress parameters	sex	G0			G1			G2			G3			p
		mean	±	median	mean	±	median	mean	±	median	mean	±	median	
TOS (nmol H_2_O_2_ Eqiv/mg)	F+M	3.92	1,41	4,08	3,95	1,52	3,85	5,08	2,39	4,82	4,88	2,12	5,02	0,005*
	F	3,37	1,02	3,27	3,98	1,17	4,24	5,3	1,51	4,92	4,61	1,68	4,69	0,001**
	M	4,19	1,55	4,38	3,98	1,73	3,82	4,63	3,72	3,35	5,1	2,45	5,14	0,09
OSI (TOS/TAC ratio)	F+M	4,16	1,47	4,48	4,31	1,64	4,26	5,50	2,77	5,22	5,25	2,30	5,19	0,006*
	F	3,58	1,14	3,54	4,29	1,2	4,56	5,68	1,65	5,29	5,0	1,83	4,96	0,001**
	M	4,43	1,59	4,6	4,37	1,89	4,13	5,14	4,43	3,8	5,45	2,67	5,54	0,14
CAT (nmolH_2_O_2_/min/mg protein)	F+M	0,13	0,09	0,07	0,17	0,16	0,13	0,21	0,19	0,14	0,28	0,46	0,13	0,17
	F	0,15	0,12	0,09	0,15	0,09	0,13	0,21	0,2	0,14	0,16	0,12	0,14	0,81
	M	0,13	0,09	0,07	0,18	0,2	0,13	0,21	0,19	0,14	0,38	0,6	0,14	0,1
SOD (mU/mg protein)	F+M	1,48	1,47	0,96	0,41	0,25	0,37	0,39	0,19	0,42	0,41	0,30	0,29	0,002*
	F	1,24	1,62	0,64	0,47	0,2	0,48	0,45	0,17	0,43	0,47	0,32	0,35	0,96
	M	1,67	1,42	1,13	0,36	0,27	0,31	0,26	0,2	0,15	0,34	0,29	0,21	0,001**
GSH (ug/mg protein)	F+M	1,96	1,41	1,71	1,48	0,76	1,24	1,49	1,15	1,11	1,17	0,68	1,13	0,04*
	F	2,02	0,91	2,24	1,26	0,38	1,2	1,13	0,35	1,01	1,23	0,86	1,09	0,036**
	M	1,98	1,67	1,54	1,6	0,91	1,48	2,27	1,8	1,42	1,12	0,54	1,22	0,23
TAC (umol/mg protein)	F+M	0,95	0,08	0,93	0,91	0,04	0,91	0,93	0,04	0,93	0,94	0,07	0,92	0,04*
	F	0,95	0,08	0,95	0,92	0,06	0,91	0,93	0,04	0,93	0,94	0,08	0,92	0,49
	M	0,98	0,08	0,92	0,91	0,03	0,91	0,94	0,07	0,93	0,94	0,06	0,91	0,16
FRAP (umol/mg protein)	F+M	0,38	0,04	0,38	0,36	0,03	0,36	0,37	0,02	0,36	0,37	0,03	0,36	0,21
	F	0,38	0,04	0,38	0,36	0,02	0,36	0,36	0,02	0,35	0,36	0,03	0,35	0,18
	M	0,39	0,04	0,38	0,36	0,04	0,36	0,39	0,02	0,39	0,37	0,03	0,37	0,059
GPx (mU/mg protein)	F+M	0,43	0,08	0,41	0,37	0,03	0,37	0,39	0,09	0,38	0,38	0,04	0,37	0,001*
	F	0,42	0,04	0,43	0,36	0,03	0,36	0,37	0,03	0,37	0,39	0,05	0,4	0,0094**
	M	0,43	0,1	0,41	0,37	0,03	0,37	0,44	0,15	0,39	0,36	0,03	0,37	0,0028**

*p<0,05 – statistically significant differences between all the study groups without division by sex.

**p<0,05 – statistically significant differences between all the study groups with division by sex.

### 3.1 Redox biomarkers (TOS, OSI)

**Figure 3:**
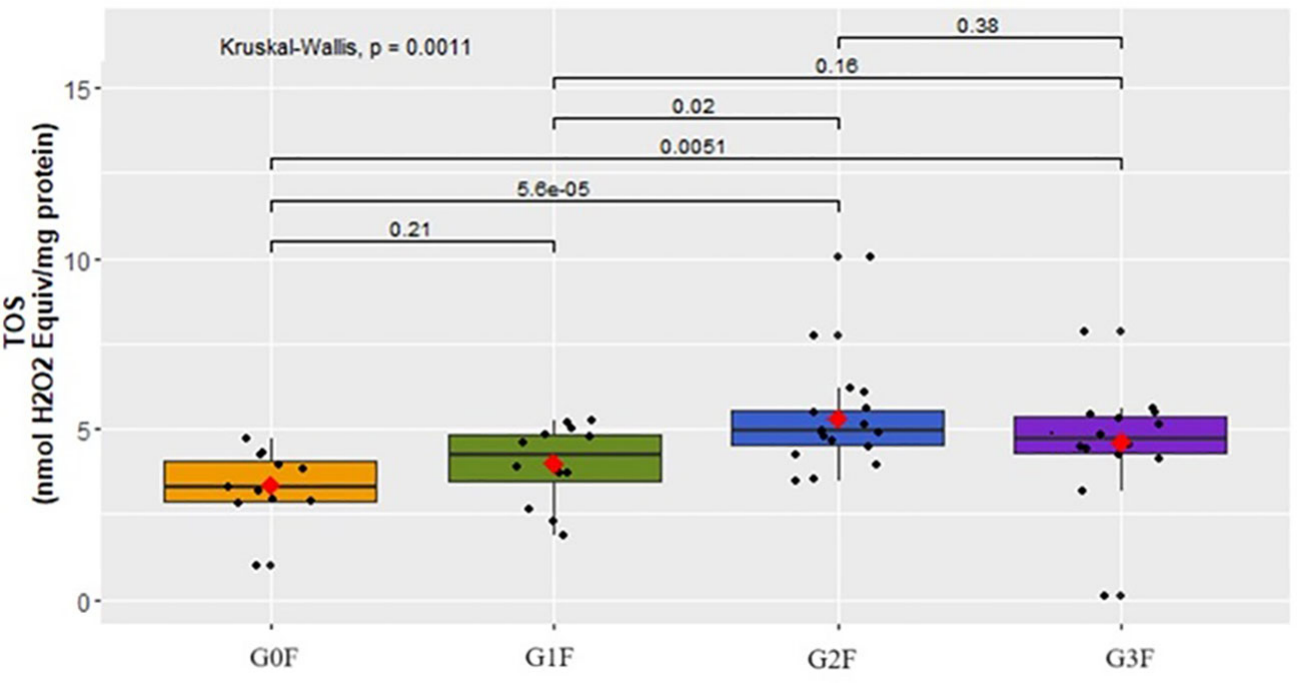
Analysis of TOS concentration (nmol H_2_O_2_ Equiv/mg protein) in the plasma of examined women in respective groups.

The female cohort with depression and schizophrenia exhibited notable distinctions in TOS concentration (p=0.0011). The findings are illustrated in **Figure 3**.

TOS concentrations increased in groups showing suicidal behavior (G1F: $\bar{x}$ =3.9 ± 1.17, G2F: $\bar{x}$ =5.3 ± 1.51, G3F: $\bar{x}$ =4.6 ± 1.68) compared to the control group (G0F: $\bar{x}$ =3.4 ± 1.02). Regarding the findings in women, a significant increase in Total Oxidant Status (TOS) levels was observed in those with suicidal thoughts and a tendency to act on them (G2F: $\bar{x}$ 5.3 ± 1.51) compared to women with suicidal thoughts but no tendency to act on them (G1F: $\bar{x}$ =3.9 ± 1.17).

**Figure 4:**
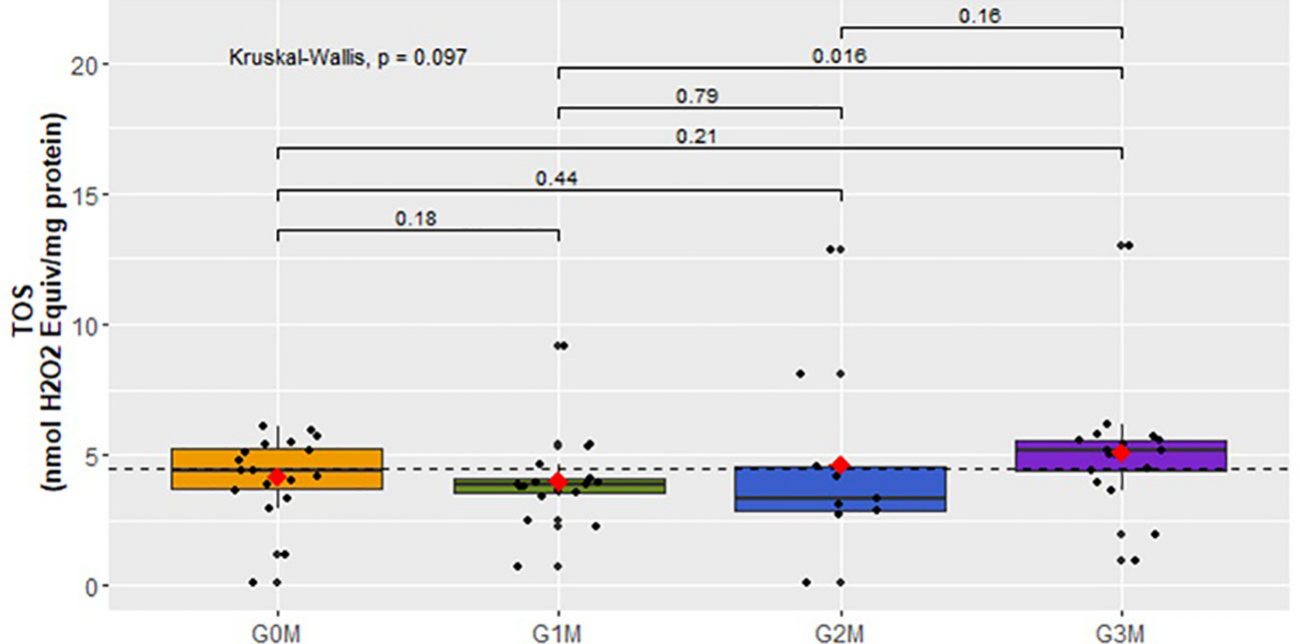
Analysis of TOS concentration (nmol H_2_O_2_ Equiv/mg protein) in the plasma of examined men in respective groups.

The analysis of TOS concentration in the male cohorts did not show statistically significant differences (**Figure 4**). Only a significant increase in TOS can be observed in the group of men after a suicide attempt (G3M: $\bar{x}$ =5.1 ± 2.45) compared to the group of men with suicidal thoughts but no tendency to act on them (G1M: $\bar{x}$ =3.98 ± 1.73).

**Figure 5:**
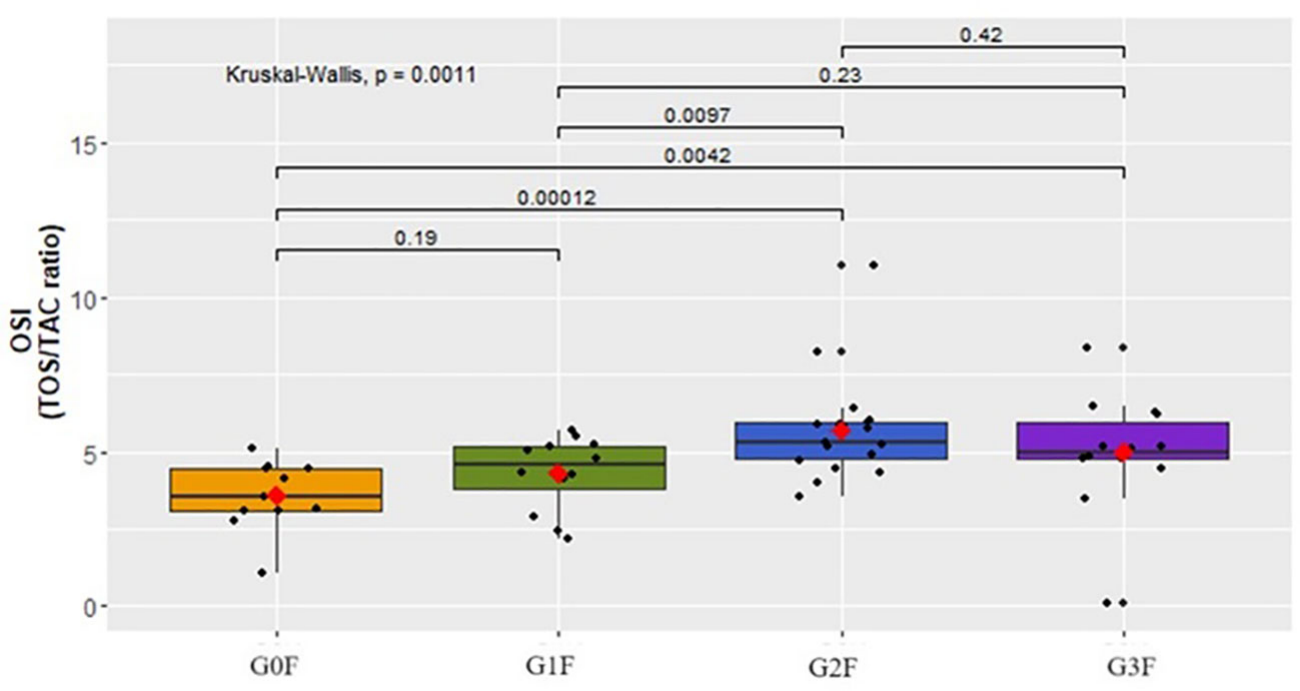
Assessment of the OSI index (TOS/TAC ratio) in the plasma of women in respective groups.

The concentration of OSI was evaluated in the female cohort, revealing statistically significant differences (**Figure 5**), with the parameter’s concentration increasing in tandem with the escalation of suicidal behaviors (p=0,001).

Among women exhibiting suicidal tendencies (G2F: $\bar{x}$ =5.7 ± 1.65, G3F: $\bar{x}$ .0 ± 1.83), a statistically significant elevation in the concentration of the OSI parameter in blood plasma was noted compared to the control group (G0F: $\bar{x}$ =3.6 ± 1.14) (**Figure 5**). Similarly, the Oxidative Stress Index (OSI) levels were significantly higher in women with suicidal thoughts and a tendency to act on them (G2F: $\bar{x}$ =5.7 ± 1.65) compared to those without such tendencies (G1F: $\bar{x}$ =4.3 ± 1.20).

**Figure 6:**
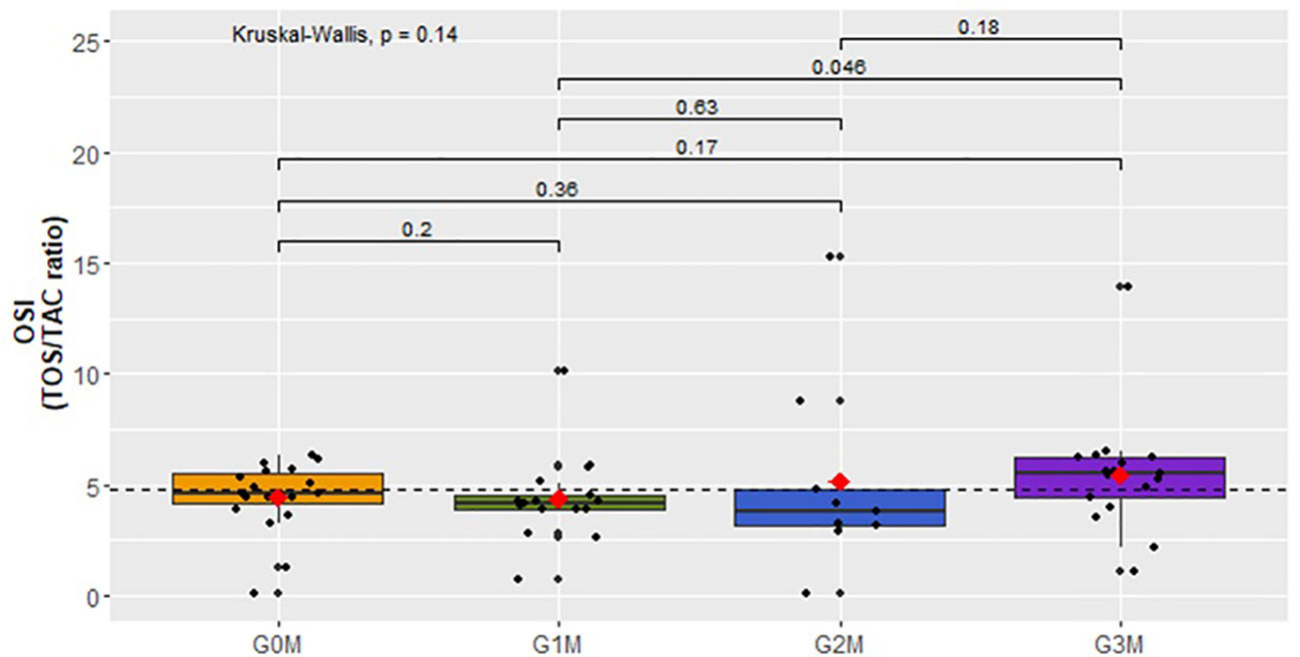
Assessment of the OSI index (TOS/TAC ratio) in the plasma of men in respective groups.

No analogous distinctions were observed within the male cohort (**Figure 6**).

### 3.2 Antioxidant parameters (GPx, SOD, GSH)

**Figure 7:**
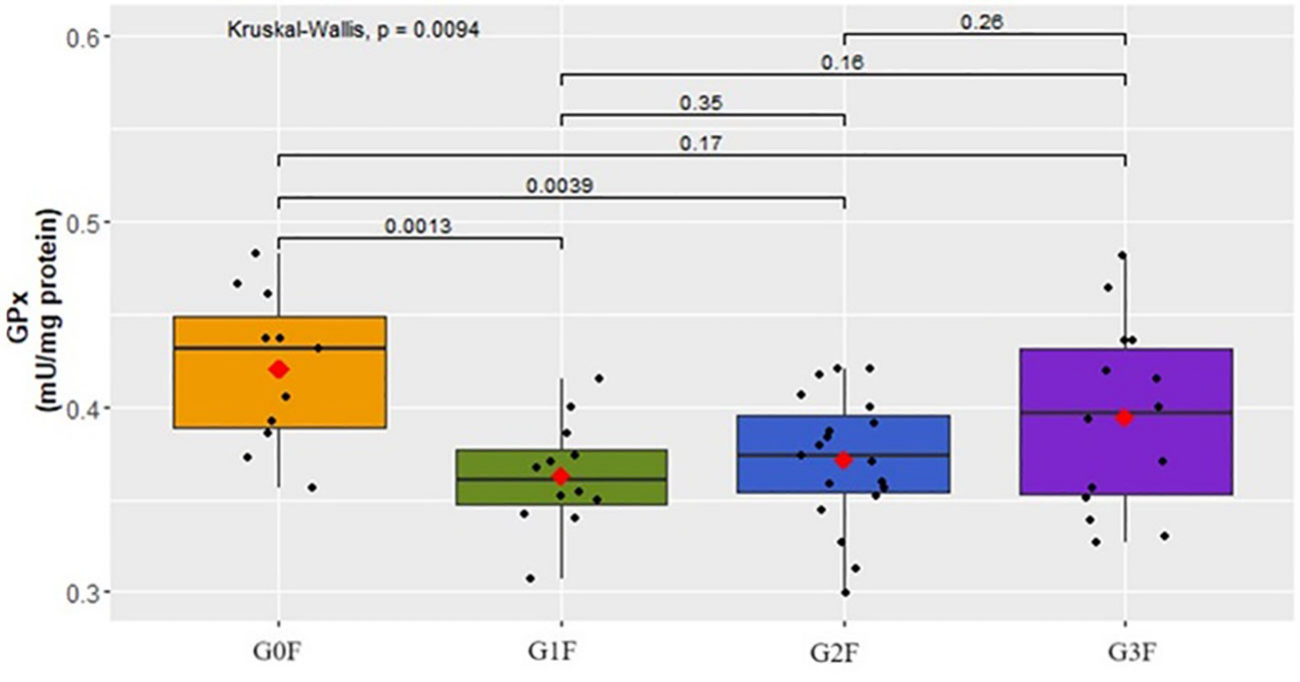
Analysis of GPx activity (mU/mg protein) in the plasma of examined women in respective groups.

**Figure 8:**
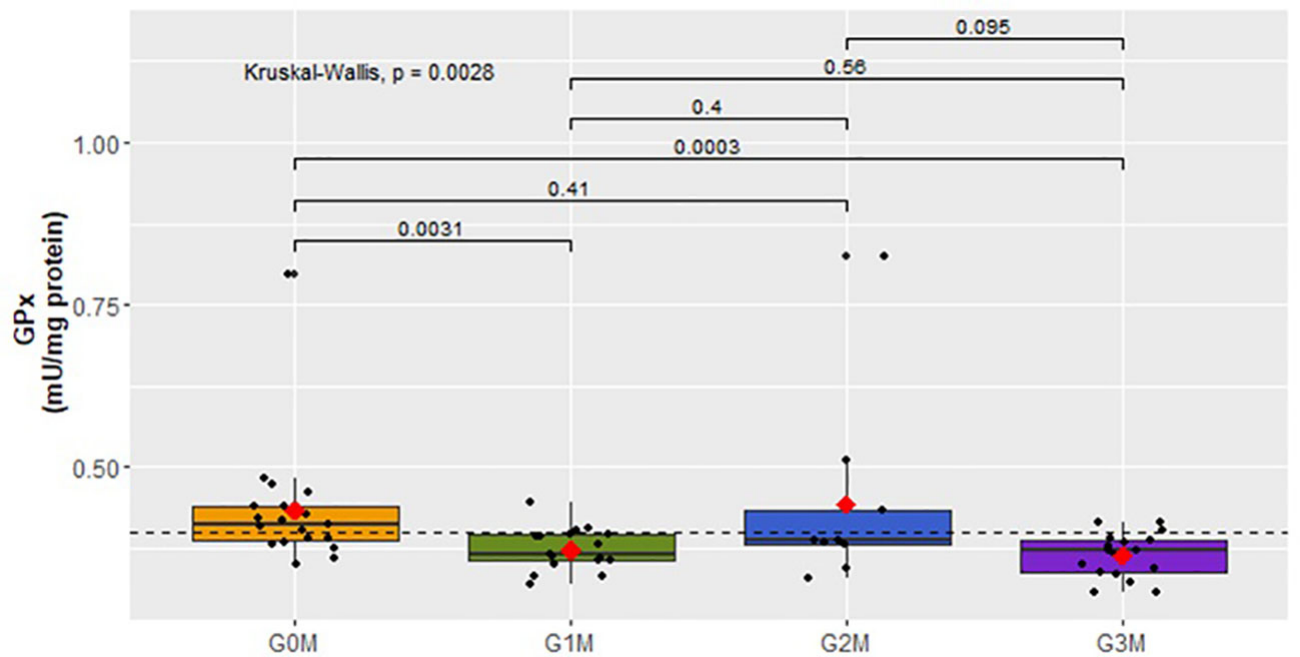
Analysis of GPx activity (mU/mg protein) in the plasma of subjects in respective groups of men.

Upon analyzing the antioxidant parameters of oxidative stress, statistically significant differences were found in the activity of the GPx parameter in women (p=0.0094) (**Figure 7**) and in men (p=0.0028) (**Figure 8**).

In the female cohorts, a decline in GPx levels was noted within the groups exhibiting suicidal tendencies (G1F: $\bar{x}$ =0.4 ± 0.03, G2F: $\bar{x}$ =0.4 ± 0.03) in comparison to the control group – G0F $\bar{x}$ (=0.4 ± 0.04) (**Figure 7**). For the male cohort, a reduction in GPx activity was observed in the G1M $\bar{x}$ (=0.4 ± 0.03) and G3M $\bar{x}$ (=0.4 ± 0.03) groups relative to the G0M control group $\bar{x}$ (=0.4 ± 0.1) (**Figure 8**).

**Figure 9:**
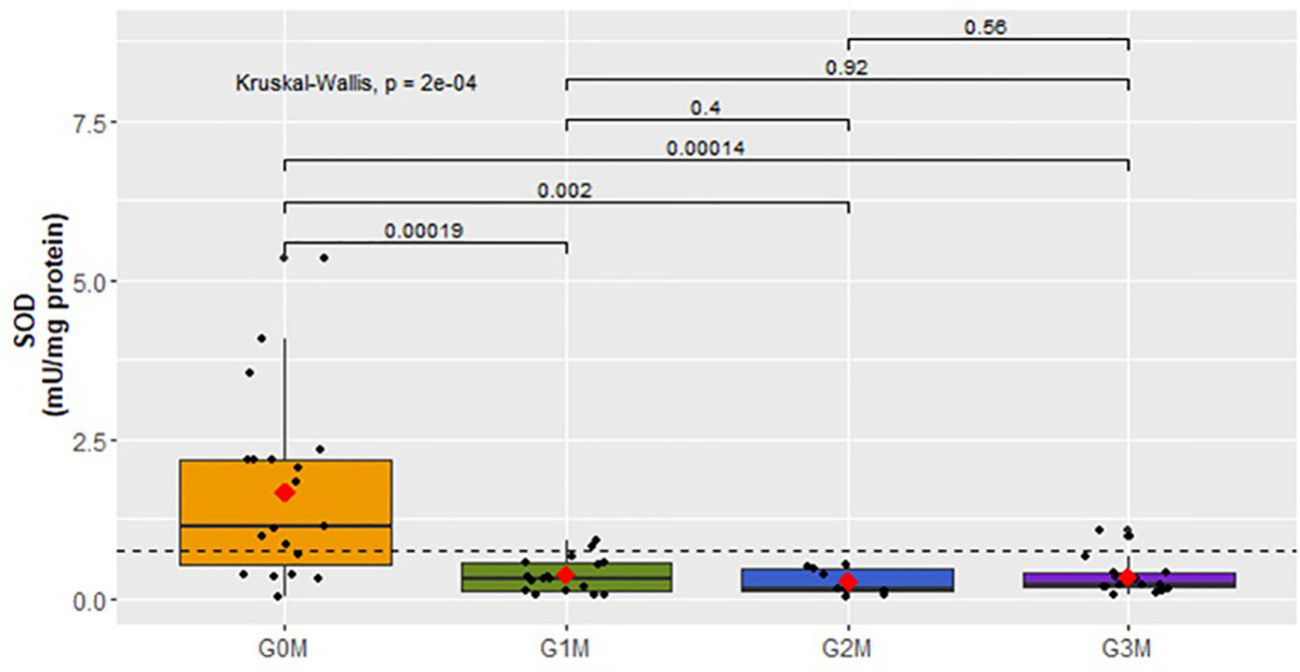
Analysis of SOD activity (mU/mg protein) in plasma in respective male cohorts.

SOD activity was also analyzed, but statistically significant results were obtained only in the male cohort (p<0,0001). The results are presented in **Figure 9**.

Among the male cohorts, a significant decrease in SOD activity was evidenced in individuals displaying suicidal behavior (G1M: $\bar{x}$ =0.4 ± 0.27), G2M: $\bar{x}$ =0.3 ± 0.20, G3M: $\bar{x}$ =0.3 ± 0.27) compared to patients in the control group (G0M) (**Figure 9**).

**Figure 10:**
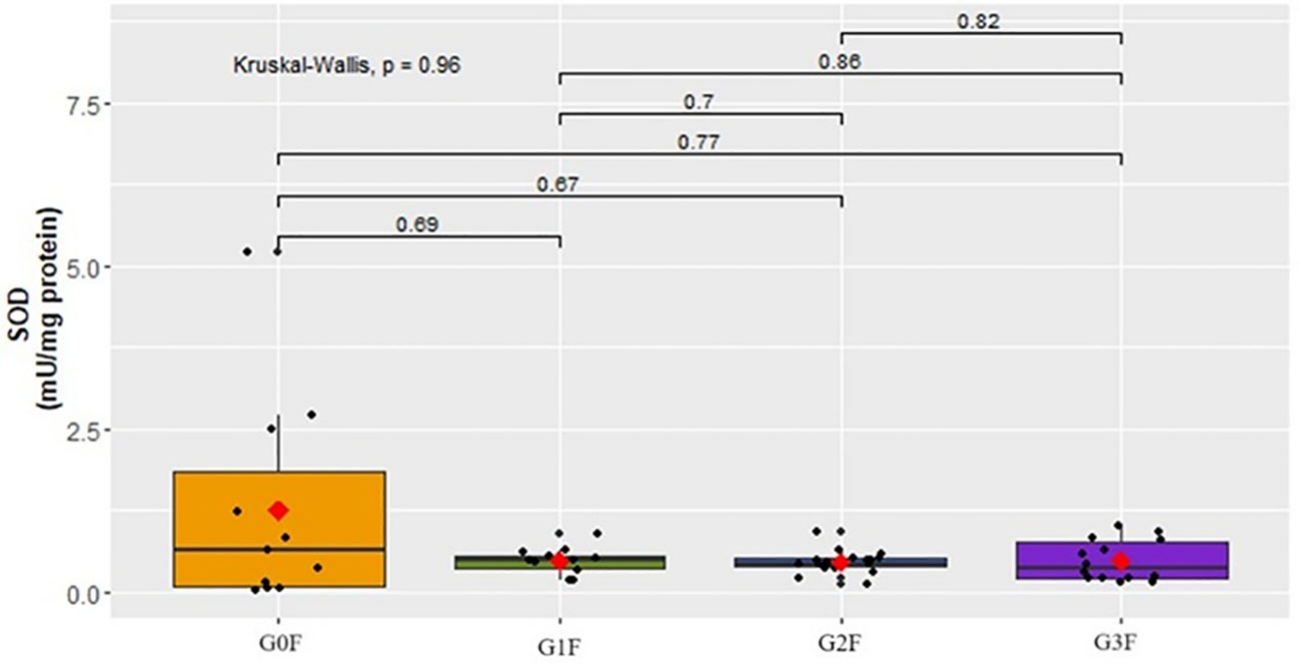
Analysis of SOD activity (mU/mg protein) in plasma in respective groups of female cohorts.

In the female cohorts, the analysis of SOD activity did not show statistically significant differences (**Figure 10**).

**Figure 11:**
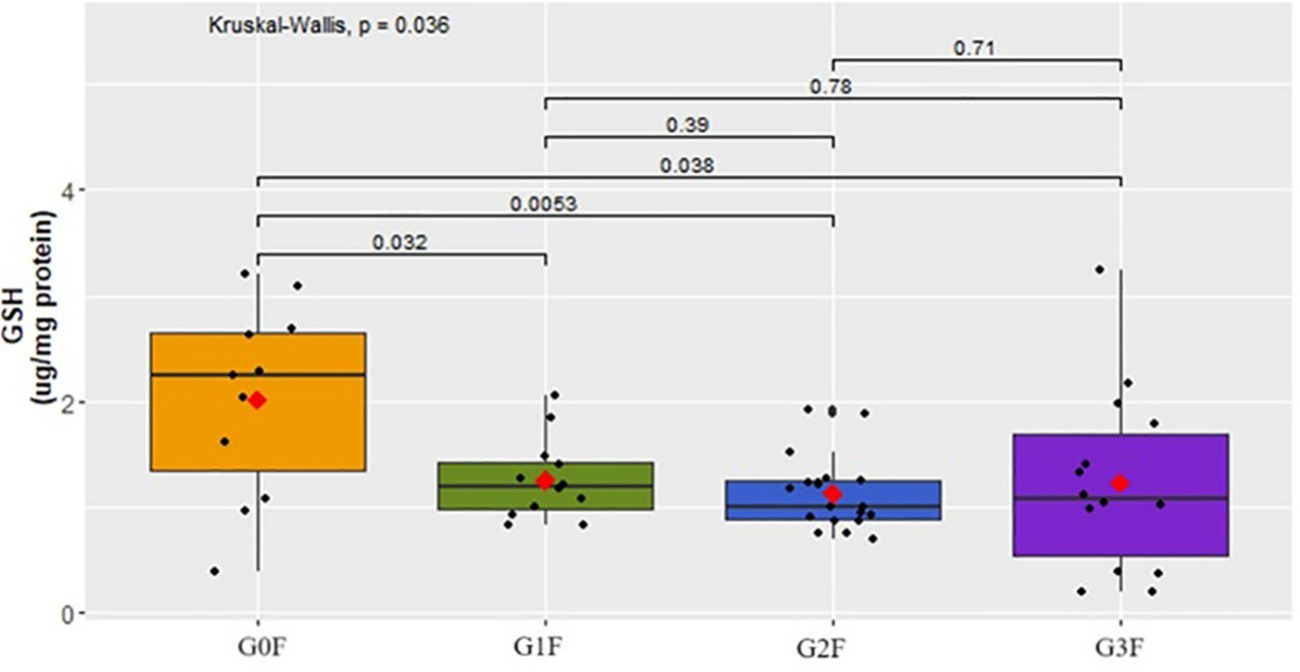
Analysis of GSH concentration (µg/mg protein) in plasma in respective groups of female cohorts.

The analysis of the results indicates significant differences between groups of women of the GSH concentration (p=0,036) as shown in **Figure 11** (women).

**Figure 12:**
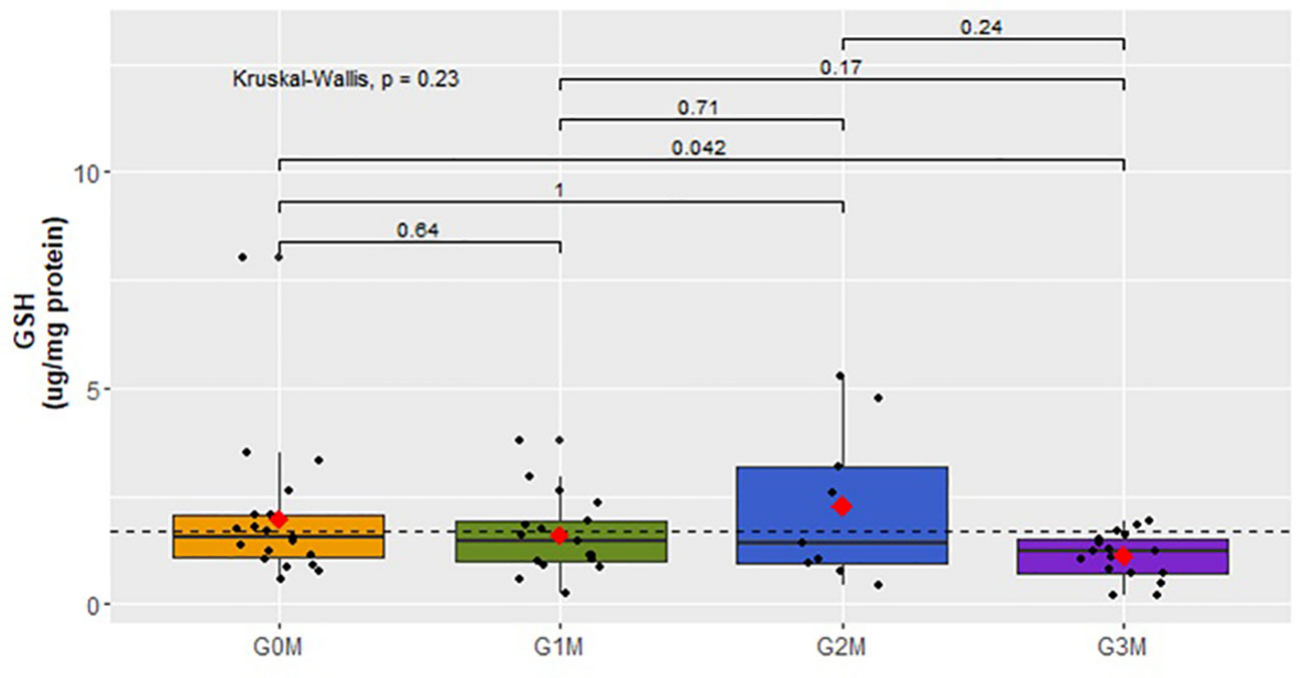
Analysis of GSH concentration (µg/mg protein) in plasma in respective male cohorts.

The examination of GSH concentration in the plasma of the female participants revealed a statistically significant decrease in the groups displaying suicidal behavior (G1F: $\bar{x}$ =1.3 ± 0.38, G2F: $\bar{x}$ =1.1 ± 0.35, G3F: $\bar{x}$ =1.2 ± 0.86) compared to the control group (G0F: $\bar{x}$ =2.0 ± 0.91) (**Figure 11**). The analysis of the results does not indicates significant differences between groups of men of the GSH concentration (p=0,23). However, only a tendency to decrease the GSH concentration was observed in the group of men after a suicide attempt (G3M: $\bar{x}$ =1.12 ± 0.54) compared to men from the control group (G0M: $\bar{x}$ =1.98 ± 1.67) (**Figure 12**).

CAT, TAC and FRAP parameters were also analyzed but no statistically significant differences were observed. The summary results are presented in **Table 2**.

## 4 Discussion

Oxidative stress factors are considered determinants of suicidal behaviors. A growing body of research is presenting evidence indicating the association between specific mental disorders (such as severe depression, autism spectrum disorders, and anxiety disorders) and irregularities in oxidative stress parameters.

Our study suggests an elevation in the levels of TOS and OSI in the plasma of surveyed women as the intensity of suicidal thoughts increases. This correlation is not evident in the group of men. However, an escalation in the concentration of TOS and OSI is noted in groups exhibiting higher levels of suicidal behavior, irrespective of gender. This finding aligns with outcomes from other studies. Koweszko et al. reported an upsurge in the OSI index among patients with suicidal thoughts over the past three months compared to those who did not report such thoughts during this period (5). While their study encompassed both women and men without distinguishing suicidal thoughts based on their likelihood of realization, our research additionally revealed an increase in OSI and TOS concentrations in the group of women with suicidal thoughts tending towards acting upon them, compared to those without such tendencies. This observation suggests a rise in these parameters in tandem with heightened suicidal tendencies.

A decrease in GPx activity in the body leads to the accumulation of reactive oxygen species (ROS) and amplifies autonomous symptoms of depression. Moreover, GPx plays a protective role against neuronal and DNA damage, as well as cell death (17). Our research, focusing on antioxidant parameters, revealed statistically significant differences in GPx activity across groups of women and men, as well as in the combined analysis without gender division. Activity reduction was observed in groups exhibiting suicidal behavior compared to the control group (without suicidal tendencies). This suggests an increased GPx metabolism in the presence of suicidal tendencies. Contrarily, Loo JL’s study did not show statistically significant differences in GPx activity in the group of patients with suicidal thoughts when compared to the control group, with no consideration for the sex of individuals with bipolar disorder (3). Similar non-significant results were obtained in the analysis of SOD activity (3). Our research, however, indicated a significant decrease in SOD activity in both the combined analysis without sex division and in the groups of men. This reduction was observed in all male groups exhibiting suicidal tendencies compared to the control group. In Vaváková’s study, involving patients of both sexes suffering from depression, the obtained results for SOD activity indicated both an increase and a decrease in this parameter’s activity in blood plasma (17). Meanwhile, Oshnokhah et al. compared the activity of NO, SOD, and TAC in blood plasma and showed a statistically significant decrease in SOD and TAC concentration and activity in the group of individuals after a suicidal attempt (7). On the other hand, the TAC concentration results in our study indicated an increase in concentration in groups with suicidal thoughts tending towards acting upon them and in the group after a suicidal attempt. However, this difference was not observed in the analysis that included separate cohorts of women and men.

The presence of major depressive disorder (MDD) is linked not only to heightened oxidative damage but also to diminished protection against oxidation. This is associated with elevated cortisol levels and reduced activity of GPx1 and SOD, leading to damage to the nerve cells. Studies by Chandley et al. underscore this correlation in patients with MDD who have died by suicide (18).

In individuals with schizophrenia, a reduction in glutathione in the prefrontal cortex and activation of oxidative stress, including GSH deficiency, have been observed (19, 20). Our study scrutinized the decline in GSH concentration in female patients exhibiting suicidal behavior. A statistically significant difference was noted in all three groups of women displaying suicidal behavior compared to the control group and in the analysis encompassing all participants collectively.

Furthermore, our study examined the CAT parameter. The findings do not reveal any statistically significant differences between the groups or in comparison to the control group of patients. However, research conducted by Loo JL indicates a statistically significant elevation in CAT concentration in the blood plasma of patients expressing suicidal thoughts compared to patients diagnosed with bipolar disorder without suicidal thoughts and to the healthy control group (3).

The research conducted by Koweszko et al. and Oshnokhah et al. did not reveal statistically significant differences in FRAP concentration among specific groups of individuals displaying suicidal behavior which is consistent with our results (5, 7).

## 5 Conclusions

- The obtained results suggest that redox biomarkers, namely TOS and OSI, in the blood plasma of women increase in tandem with the severity of suicidal behavior, encompassing both thoughts and attempts. The observation of an elevation in the concentration of these indicators might aid in distinguishing women who genuinely contemplate suicide from those who may not be inclined to act on their expressed suicidal intentions.
- Antioxidant parameters that decline with the onset of suicidal behaviors (thoughts and attempts) may only signify the presence of these tendencies in a given patient, as the observed variations in the concentrations and activity of these parameters were significant solely in comparison to the control group. No notable alterations in SOD, GPx, and GSH concentrations and activity were noted between groups exhibiting suicidal behavior.
- Redox biomarkers TOS and OSI could prove valuable in diagnosing women at a genuine risk of committing suicide. On the other hand, antioxidant parameters – SOD, GPx, and GSH may be instrumental in identifying patients with suicidal behaviors, without specifying their intensity.

## Data availability statement

The raw data supporting the conclusions of this article will be made available by the authors, without undue reservation.

## Ethics statement

The studies involving humans were approved by the local Bioethics Committee of the Medical University of Białystok, Poland (No: R-I-002/33/2016). The studies were conducted in accordance with the local legislation and institutional requirements. The participants provided their written informed consent to participate in this study.

## Author contributions

ML: Data curation, Resources, Writing – original draft, Writing – review & editing. LO: Conceptualization, Supervision, Writing – original draft, Writing – review & editing. NW: Conceptualization, Supervision, Writing – review & editing. AK-B: Data curation, Investigation, Resources, Writing – review & editing. MM: Methodology, Supervision, Writing – review & editing. KW-S: Resources, Writing – review & editing. AZ: Methodology, Writing – review & editing. KD: Methodology, Writing – review & editing. MŻ-P: Supervision, Writing – review & editing.
